# Multimodal remote sensing benchmark datasets for land cover classification with a shared and specific feature learning model

**DOI:** 10.1016/j.isprsjprs.2021.05.011

**Published:** 2021-08

**Authors:** Danfeng Hong, Jingliang Hu, Jing Yao, Jocelyn Chanussot, Xiao Xiang Zhu

**Affiliations:** a Remote Sensing Technology Institute, German Aerospace Center, 82234 Wessling, Germany; b Data Science in Earth Observation, Technical University of Munich, 80333 Munich, Germany; c Aerospace Information Research Institute, Chinese Academy of Sciences, 100094 Beijing, China; d Univ. Grenoble Alpes, INRIA, CNRS, Grenoble INP, LJK, 38000 Grenoble, France

**Keywords:** Benchmark datasets, Classification, Feature learning, Hyperspectral, Land cover mapping, DSM, Multimodal, Multispectral, Remote sensing, SAR, Shared features, Specific features

## Abstract

As remote sensing (RS) data obtained from different sensors become available largely and openly, multimodal data processing and analysis techniques have been garnering increasing interest in the RS and geoscience community. However, due to the gap between different modalities in terms of imaging sensors, resolutions, and contents, embedding their complementary information into a consistent, compact, accurate, and discriminative representation, to a great extent, remains challenging. To this end, we propose a shared and specific feature learning (S2FL) model. S2FL is capable of decomposing multimodal RS data into modality-shared and modality-specific components, enabling the information blending of multi-modalities more effectively, particularly for heterogeneous data sources. Moreover, to better assess multimodal baselines and the newly-proposed S2FL model, three multimodal RS benchmark datasets, i.e., *Houston2013* – hyperspectral and multispectral data, *Berlin* – hyperspectral and synthetic aperture radar (SAR) data, *Augsburg* – hyperspectral, SAR, and digital surface model (DSM) data, are released and used for land cover classification. Extensive experiments conducted on the three datasets demonstrate the superiority and advancement of our S2FL model in the task of land cover classification in comparison with previously-proposed state-of-the-art baselines. Furthermore, the baseline codes and datasets used in this paper will be made available freely at https://github.com/danfenghong/ISPRS_S2FL.

## 1 Introduction

The rapid development of remotely sensed imaging techniques enables the measurement and monitoring of Earth on the land surface and beneath (e.g., identification of underground minerals (Bishop et al., 2011), geological environment survey and monitoring (Van der Meer et al., 2012), volcanic terrain component analysis (Amici et al., 2013)), of the quality of air and water, and of the health of humans, plants, and animals (Nativi et al., 2015). Remote sensing (RS) is one of the most important contact-free sensing means for Earth observation (EO) to extract relevant information about the physical properties of the Earth and environment system from spaceborne and airborne platforms. With the ever-growing availability of RS data sources from both satellite and airborne sensors on a large scale and even global scale, multimodal RS image processing and analysis techniques have been garnering growing attention in various EO-related tasks(Schmitt and Zhu, 2016), such as land cover change detection (Liu et al., 2017, Liu et al., 2019), disaster monitoring and management (Zhu et al., 2019, Liu et al., 2020), urban planning (Weng, 2009, Xie and Weng, 2017), mineral exploration (Hong et al., 2019b, Siebels et al., 2020).

The data acquired by different platforms can provide diverse and complementary information, including light detection and ranging (LiDAR) or digital surface model (DSM) providing the height information about the ground elevation, synthetic aperture radar (SAR) providing the structure information about Earth’s surface, and multispectral (MS) or hyperspectral (HS) data providing detailed content information of sensed materials. The joint exploitation of different RS data has been therefore proven to be useful to further enhance the understanding, possibilities, and capabilities to Earth and our environment. In a complex urban scene, the ability of spectral data (e.g., RGB, MS, HS) in finely identifying the land cover categories usually remains limited, particularly for those categories that have extremely similar spatial structure or spectral signatures. For example, the material “Asphalt” on the road or on the roof can be hardly classified by only observing subtle discrepancies from their spectral profiles (Heiden et al., 2012). But fortunately, we might expect to have the height information provided by DSM data, which enables the fine-grained recognition of these similar materials at a higher accuracy compared to only single modalities. It is well known that HS images are characterized by nearly continuous spectral properties, while MS images can provide finer spatial information. The fusion of MS and HS images naturally becomes a feasible solution to obtain the image product of high spatial and spectral resolutions. Another common example is cloud removal. Optical RS data (e.g., RGB, MS, HS) tend to suffer from the cloud occlusion in imaging process, thereby bringing the risk of important information loss. SAR data or DSM data generated from the LiDAR are insensitive to the cloud coverage by capturing intrinsic structure or elevation information that are not closely associated with the cloud (Gao et al., 2020).

In recent years, many previous works have been proposed by the attempts to boost the development of multimodal RS techniques in land cover classification. Nevertheless, the ability of these approaches in multimodal RS data representations remains limited, further limiting the performance gain of subsequent high-level applications. This can be well explained by two possible factors as follows.

• **Multimodal RS Benchmark Datasets.** Despite the increased availability of the multimodal RS data, different contexts, structures, sensors, resolutions, and imaging conditions pose a great challenge in data acquisition and processing for to-be-studied scenes (Dalla Mura et al., 2015). Consequently, the lack of multimodal RS benchmark datasets, to a larger extent, limits the development of the corresponding methodologies and the practical application of land cover classification.
• **Multimodal Feature Learning Models.** Feature extraction (FE), as one of the most important steps prior to the high-level data analysis, also plays a key role in multimodal RS. Most of previous FE methods, either hand-crafted or learning-based (Rasti et al., 2020), usually extract or learn multimodal features in a concatenation fashion. Such a manner could lead to inadequate information fusion and even hurt or destroy the original components of each modality, due to the highly coupled information between multimodal RS data (particularly heterogeneous data).

According to the aforementioned challenges, we aim in this paper to build diversified multimodal RS benchmark datasets and devise novel feature learning models for land cover classification. For this purpose, we first collect multiple RS data with different resolutions, different modalities, and different sensors, with well-labeled land cover maps. Supported by these datasets, we propose a novel multimodal feature learning (MFL) model by decomposing different modalities into shared and specific representations, in order to fuse diverse information from multimodal RS data in a compacted and discriminative way. The proposed model establishes the explicit mapping relations between the to-be-learned features and original multimodal RS data, providing an interpretable MFL model. More specifically, the contributions of this paper can be highlighted as follows.

• Three multimodal RS benchmark datasets are prepared and built with the application to land cover classification. They are diversified, including homogeneous HS-MS *Houston2013* datasets, heterogeneous HS-SAR *Berlin* datasets, and heterogeneous HS-SAR-DSM *Augsburg* datasets. We will make them freely and openly available after a possible publication, contributing to the RS and information fusion communities. Currently, the heterogeneous RS feature learning and fusion techniques are less investigated, particularly three-modality datasets. To our best knowledge, this is the first time to open heterogeneous RS benchmark datasets that simultaneously involve HS, SAR, and DSM data for land cover classification.
• A shared and specific feature learning (S2FL) model is devised to extract diagnostic features from multimodal RS data. S2FL is capable of decoupling different modalities into shared and specific feature spaces, respectively, by aligning the common components between multi-modalities on manifolds. Such separable properties tend to capture fine-grained differences between different categories, further yielding better classification results.
• An alternating direction method of multipliers (ADMM)-based optimization framework is customized for the fast and accurate solutions of the proposed S2FL model.

The rest of this paper is organized as follows. In Section 2, a deep literature review is made in terms of related work in MFL. Section 3 then elaborates on the methodology of our proposed S2FL model as well as the ADMM-based model optimization process. In Section 4, extensive experiments are conducted on three multimodal RS benchmarks in comparison with several state-of-the-art baselines. Finally, Section 5 makes the summary with some important conclusions and hints at potential future research trends.

## 2 Related work

Image-level fusion is a straightforward way to enhance certain information (e.g., spatial or spectral resolutions) of homogeneous data, such as RS image pansharpening (Ehlers et al., 2010), HS and MS fusion (Wei et al., 2015). However, there are more heterogeneous RS data in reality, typically optical and SAR data that can provide richer and more complementary information. The image-level fusion can not meet the demand for the heterogeneous data fusion task to some extent. Feature-level learning and fusion are needed. For decades, extensive efforts have been made by researchers to develop a variety of MFL algorithms for land cover classification of RS data. These existing methods can be roughly categorized into two main groups from the perspectives of different fusion strategies, i.e., concatenation-based MFL and alignment-based MFL.

### 2.1 Concatenation-based MFL models

As the name suggests, concatenation-based MFL models can obtain the fused features by

• first stacking the input multimodal RS images and then passing through a certain feature extractor or learner;
• or first extracting or learning the feature representations for each modality and then stacking them as a certain classifier input.

There have been many classic and state-of-the-art models related to concatenation-based MFL in RS. Morphological operators (Fauvel et al., 2008), as the main member of the feature extractor family, have been widely and successfully applied to multimodal RS image feature extraction and classification. For instance, Liao et al. generalized the graph embedding model (Hong et al., 2020b) for the fusion of the morphological profiles of HS and LiDAR data in land cover classification (Liao et al., 2014). In (Rasti et al., 2017), a novel component analysis model based on total variation was designed to further refine the feature representations of extinction profiles (Fang et al., 2017) obtained from HS and LiDAR data. Yokoya *et al.* extracted morphological features of time-series MS data and corresponding OpenStreetMaps and concatenated them as the classifier input (Yokoya et al., 2017). Ma *et al.* used the multisource RS data for “Ghost City” phenomenon identification (Ma et al., 2018). Authors of (Chen et al., 2017) proposed to fuse the multisource RS data by the means of deep networks for accurate land cover classification. Ref. (Xia et al., 2019) developed a semi-supervised graph fusion model for HS and LiDAR data classification. Inspired by the recent advancement of deep learning techniques in data representations, enormous learning-based approaches have been developed for multimodal RS feature fusion(Zhu et al., 2017). (Hong et al., 2021) for the first time proposed a general and unified deep learning framework for multimodal RS image classification. In (Hang et al., 2020), a coupled convolutional neural network was employed to fuse the heterogeneous RS data in the feature level. However, there is room for improvement in the interpretablity of DL-based methods. The interpretable knowledge embedding can guide the network learning towards better solutions (or results) more effectively. Furthermore, the past decades have witnessed a favorable development of concatenation-based MFL in the RS community, yet the ability to fully take advantage of the diverse information of different modalities, especially heterogeneous data, remains limited.

### 2.2 Alignment-based MFL models

Unlike the above feature concatenation strategy, alignment-based MFL methods seek to learn a common feature set that multiple modalities share by the means of well-known manifold alignment (MA) techniques (Wang and Mahadevan, 2011). By introducing the MA into the RS applications, Tuia et al. aligned the multi-view MS data to a consistent representation in a semi-supervised way (Tuia et al., 2014). (Tuia and Camps-Valls, 2016) projected the multimodal data to a higher-dimensional kernel space, where different data sources can be better aligned. Moreover, the semi-supervised model in (Tuia et al., 2014) was improved by using a mapper-induced graph structure for the fusion of optical image and polarimetric SAR data (Hu et al., 2019). Inspired by the MA idea, Hong *et al.* proposed a MA-regularized representation learning model, simultaneously involving subspace learning and ridge regression, CoSpace for short (Hong et al., 2019a). CoSpace is capable of learning the alignment representations across multi-modalities, thereby yielding more effective information fusion. The same investigators further extended the CoSpace model by replacing ridge regression with sparse regression, generating a $\ell_{1}$-norm version of the CoSpace model, i.e., $\ell_{1}$-CoSpace (Hong et al., 2020a). Pournemat *et al.* (Pournemat et al., 2020) proposed a semi-supervised charting approach for multimodal MA in the spectral domain. There are also some MA-based variants successfully applied to other RS-related applications, such as visualization (Liao et al., 2016), dimensionality reduction, cross-modality retrieval (Hong et al., 2019c). Admittedly, the alignment-based strategy is capable of performing well in heterogeneous data fusion by the means of information-sharing mechanism. Yet only capturing the shared information across multi-modalities is hardly achievable to learn better multimodal feature representations, due to the lack of modeling or fusing modality-specific properties.

## 3 S2FL: Shared and specific feature learning model

### 3.1 Method overview

**Fig. 1:**
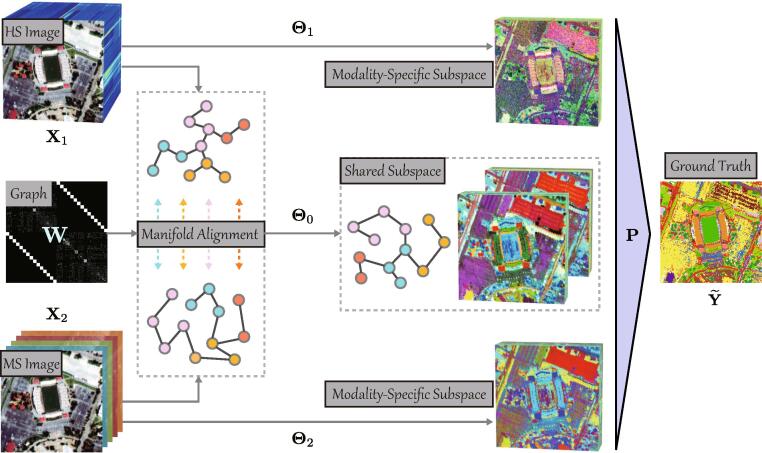
An illustration to clarify the learning process for shared and specific subspaces (or features) of multimodal RS data in the proposed S2FL model. The to-be-estimated variables $\Theta_{0},{\left\{\Theta_{k}\right\}}_{k=1}^{2}$, and P denote the shared subspace projection, the specific subspace projections, and the regression matrix, respectively, Note that we here take the bi-modality as an example.

To enhance the representation ability of multimodal data fusion and reduce the information loss (possibly due to only considering modality-shared properties and ignoring those modality-specific ones), we seek to find a more discriminative feature space by disentangling different data sources into shared and specific domains. Using the to-be-learned features, a better decision boundary is expect to obtain in the classification task. For this purpose, a shared and specific feature learning model is devised, called S2FL, by aligning shared components between multi-modalities on the latent manifold subspace and simultaneously separating out their specific information. Such a modeling strategy is interpretable and effective for learning multimodal RS feature representations, further yielding the great potentials in land cover classification. Fig. 1 illustrates the flowchart of the proposed S2FL model.

### 3.2 Notation

Let $X_{k}\in R^{d_{k}\times N}$ be the unfolded matrix with respect to the $k^{\mathrm{th}}$ modality with $d_{k}$ channels by *N* pixels, and *K* be the number of all considered modalities. $Y\in R^{C\times N}$ denotes the one-hot encoding label matrix, where *C* is the number of categories. $\Theta_{0}\in R^{d_{s}\times \sum_{k=1}^{K}d_{k}}$ and $\Theta_{k}\in R^{d_{s}\times d_{k}}$ denote the shared subspace projection and specific subspace projections with the respect to the $k^{\mathrm{th}}$ modality, respectively, and $d_{s}$ means the feature (or subspace) dimension. $P\in R^{C\times d_{s}}$ is defined as the regression matrix that connects the subspace and label information (i.e., Y). $I,||X|{|}_{F}$, and (X) denote the identity matrix, the Frobenius norm of the matrix X, and the trace of the matrix X, respectively. Moreover, the Laplacian matrix is denoted by L, which can be computed by D-W. W is the adjacency matrix of X, and D is a diagonal matrix, whose diagonal elements can be obtained by $D_{\mathrm{ii}}=\sum_{i\neq j}W_{i,j}$. Table 1 gives the detailed definitions of these variables used in the S2FL model.

**Table 1:** The symbols of variables used in the proposed S2FL model as well as their description and size, where we take the bi-modality as an example, i.e., K=2.

Symbols	Description	Size
$d_{s}$	the dimension of the feature space or subspace	1×1
$d_{k}$	the dimension of the $k^{\mathrm{th}}$ modality	1×1
*C*	the number of categories	1×1
*N*	the number of pixels (or samples)	1×1
*K*	the number of considered modalities	1×1
$\cdot_{i,j}$	the ${\left(i,j\right)}^{\mathrm{th}}$ entry of the matrix ·	1×1
$X_{k}$	the unfolded matrix of the $k^{\mathrm{th}}$ modality	$d_{k}\times N$
Y	the one-hot encoding label matrix	C×N
$\Theta_{0}$	the shared subspace projections for all considered modalities	$d_{s}\times \sum_{k=1}^{K}d_{k}$
$\Theta_{k}$	the specific subspace projection for the $k^{\mathrm{th}}$ modality	$d_{s}\times d_{k}$
Θ	the generalized subspace projections, obtained by $\Theta_{0}+[\Theta_{1},\Theta_{2}]$	$d_{s}\times \sum_{k=1}^{K}d_{k}$
P	the linear regression matrix	$C\times d_{s}$
W	the adjacency matrix of to-be-aligned modalities	2N×2N
D	the degree matrix of the matrix W, obtained by $D_{\mathrm{ii}}=\sum_{i\neq j}W_{i,j}$	2N×2N
L	the Laplacian matrix of the matrix W, obtained by D-W	2N×2N
I	the identity matrix	$d_{s}\times d_{k}$
$||X|{|}_{F}$	the Frobenius norm of the matrix X, obtained by $\sqrt{\sum_{i,j}X_{i,j}^{2}}$	1×1
(X)	the trace of the matrix X	1×1

### 3.3 Problem formulation

With the aforementioned problem statement and given definitions of variables, we model the S2FL’s problem as follows

(1) $$\begin{array}{cc} & \underset{P,\Theta_{0},{\left\{\Theta_{k}\right\}}_{k=1}^{K}}{\min}\frac{1}{2}||\overset{\sim }{Y}-P\Theta \overset{\sim }{X}|{|}_{F}^{2}+\frac{\alpha }{2}||P|{|}_{F}^{2}+\frac{\beta }{2}(\Theta_{0}\overset{\sim }{X}L{\left(\Theta_{0}\overset{\sim }{X}\right)}^{⊤})\\ & \;s.t.\;\;\Theta_{k}\Theta_{k}^{⊤}=I,\;\;k=0,1,2,\ldots K, \end{array}$$

where

$$\overset{\sim }{Y}=\left[\begin{array}{ccc} Y_{1}, & \cdots , & Y_{K} \end{array}\right],\;\;\overset{\sim }{X}=\left[\begin{array}{ccc} X_{1}, & \cdots , & 0\\ \vdots & \ddots & \vdots \\ 0, & \cdots , & X_{K} \end{array}\right],\;\;\Theta =\Theta_{0}+\left[\begin{array}{ccc} \Theta_{1}, & \cdots , & \Theta_{K} \end{array}\right]\text{.}$$

α and β denote the penalty parameters to balance the importance of different terms. Please note that $Y_{1},Y_{2},\ldots$, and $Y_{K}$ only aim to show the groups from 1 to *K* corresponding to different modalities, and actually represent the same labels, i.e., Y.

**Fig. 2:**
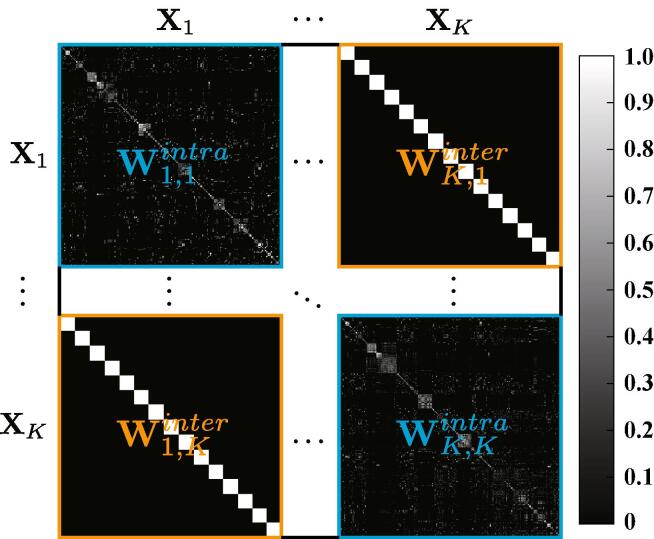
An example to illustrate the adjacency matrix (W).
• the orthogonal constraints with respect to the variables $\Theta_{0}$ and ${\left\{\Theta_{k}\right\}}_{k=1}^{K}$ are added in S2FL model to reduce the freedoms and shrink the solution space effectively, thereby finding better local optimal solutions.

The optimization problem of the first term in Eq. (1) is highly ill-posed due to its large freedom degrees (e.g., simultaneous estimation of coupled variables P and Θ). To this end,

• we regularize the variable P with its Frobenius norm, denoted as $||P|{|}_{F}$, in order to stabilize the convergence process and improve the generalization ability of the model;
• the modality-shared and modality-specific components can be learned and separated on a latent subspace by the means of one common projection $\Theta_{0}$ for all modalities and *K* characteristic projections ${\left\{\Theta_{k}\right\}}_{k=1}^{K}$ for each individual modality. This information-sharing process can be performed by using the MA regularization, i.e., $\left(\Theta_{0}\overset{\sim }{X}L{\left(\Theta_{0}\overset{\sim }{X}\right)}^{⊤}\right)$, and then the remaining information is naturally irrelevant and unique between different modalities. Moreover, the joint graph W in L consists of $k^{2}$ subgraphs, as illustrated in Fig. 2. In W, the block diagonal matrix is the intra-modality subgraph, which can be obtained by using the Gaussian kernel function with the width of σ as follows
  (2) $$W_{i,j}^{\mathrm{intra}}=\exp(-\frac{||x_{i}-x_{j}|{|}^{2}}{\sigma^{2}}),$$
  and the rest is the inter-modality subgraph, which can be directly constructed by the means of label information, leading to the following discriminative graph structure (Hong et al., 2019a):
  (3) $$W_{i,j}^{\mathrm{inter}}=\left\{\begin{array}{cc} 1/N_{C},\; & \mathrm{if}\;x_{i}\;\mathrm{and}\;x_{j}\;\mathrm{belong}\;\mathrm{to}\;\mathrm{the}\;C^{\mathrm{th}}\mathrm{class}\text{;}\\ 0,\; & \mathrm{otherwise}, \end{array}\right)$$
  where $N_{C}$ is the number of samples for the $C^{\mathrm{th}}$ class;

Algorithm 1 S2FL: Global optimization

**Table:** 

**Require:**$\overset{\sim }{Y},\overset{\sim }{X},L$, and parameters α,β,σ, and maxIter.
1. Initialize the parameter Θ using locality preserving projections (LPP) (He and Niyogi, 2004) and the parameter $\Theta_{0}=0$. The iteration starts with t=1 and the tolerate error is set to $\zeta =10^{-4}$.
2. Compute the adjacency matrix W using Eqs. (2), (3) Laplacian matrix L.
3. **for** The iteration number t=1 to maxIter**do**
4. Learn the linear regression matrix P using Eq. (5).
5. Learn the shared subspace projection matrix $\Theta_{0}$ by Algorithm 2.
6. **for**k=1 to *K***do**
7. Learn the specific subspace projection $\Theta_{k}$ for the $k^{\mathrm{th}}$ modality.
8. **end for**
9. When t>1 and calculate the loss of the objective function in the $t^{\mathrm{th}}$ and ${\left(t-1\right)}^{\mathrm{th}}$ iterations, denoted as $E^{t}$ and $E^{t-1}$, and check the stopping condition:
10. **if**$|\frac{E^{t}-E^{t-1}}{E^{t-1}}|<\zeta$**then**
11. Stop iteration.
12. **else**
13. t←t+1.
14. **end if**
15. **end for**
16. **Ensure:** Linear regression matrix P, shared subspace projection $\Theta_{0}$ for all considered modalities, and modality-specific subspace projections ${\left\{\Theta_{k}\right\}}_{k=1}^{K}$.

### 3.4 Model optimization

The optimization problem of Eq. (1) is typically non-convex, whose global minimum is usually hard to be found. We, however, expect to have local optimal solutions by alternatively optimizing separable convex subproblems of to-be-estimated variables $P,\Theta_{0}$, and ${\left\{\Theta_{k}\right\}}_{k=1}^{K}$. Algorithm 1 details an overview optimization process of the proposed S2FL model.

#### 3.4.1 Learning linear regression matrix — P

The optimization with respect to the variable P is nothing but a least-square regression problem with a common Tikhonov-Phillips regularization. This subproblem can be then written as

(4) $$\underset{P}{\min}\frac{1}{2}||\overset{\sim }{Y}-P\Theta \overset{\sim }{X}|{|}_{F}^{2}+\frac{\alpha }{2}||P|{|}_{F}^{2},$$

which has the following analytical solution

(5) $$P\leftarrow (\overset{\sim }{Y}{\overset{\sim }{X}}^{⊤}\Theta^{⊤}){\left(\Theta \overset{\sim }{X}{\overset{\sim }{X}}^{⊤}\Theta^{⊤}+\alpha I\right)}^{-1}\text{.}$$

#### 3.4.2 Learning shared subspace projection matrix — $\Theta_{0}$

The constraint optimization problem with respect to the variable $\Theta_{0}$ can be formulated as follows

(6) $$\underset{\Theta_{0}}{\min}\frac{1}{2}||\overset{\sim }{Y}-P\Theta \overset{\sim }{X}|{|}_{F}^{2}+\frac{\beta }{2}(\Theta_{0}\overset{\sim }{X}L{\left(\Theta_{0}\overset{\sim }{X}\right)}^{⊤})\;\;s.t.\;\;\Theta_{0}\Theta_{0}^{⊤}=I\text{.}$$

The problem (6) can be optimized by designing an ADMM-based solver (Boyd et al., 2011). More specifically, two auxiliary variables H and G are introduced into the Eq. (6) to replace $\Theta_{0}\overset{\sim }{X}$ in the first term and $\Theta_{0}$ in the constraint term, respectively, we then have the following equivalent form of Eq. (6)

(7) $$\underset{\Theta_{0},H,G}{\min}\frac{1}{2}||\overset{\sim }{Y}-P[\Theta_{1},\cdots ,\Theta_{K}]\overset{\sim }{X}-\mathrm{PH}|{|}_{F}^{2}+\frac{\beta }{2}(\Theta_{0}\overset{\sim }{X}L{\left(\Theta_{0}\overset{\sim }{X}\right)}^{⊤})$$

$$s.t.\;\;H=\Theta_{0}\overset{\sim }{X},\;\;G=\Theta_{0},\;\;{\mathrm{GG}}^{⊤}=I\text{.}$$

We further rewrite the Eq. (6) to its augmented Lagrangian function:

(8) $$\begin{array}{cc} L & \left(\Theta_{0},H,G,\Lambda_{1},\Lambda_{2}\right)\\ & =\frac{1}{2}||\overset{\sim }{Y}-P[\Theta_{1},\cdots ,\Theta_{K}]\overset{\sim }{X}-\mathrm{PH}|{|}_{F}^{2}+\frac{\beta }{2}(\Theta_{0}\overset{\sim }{X}L{\left(\Theta_{0}\overset{\sim }{X}\right)}^{⊤})\\ & +\Lambda_{1}^{⊤}(H-\Theta_{0}\overset{\sim }{X})+\Lambda_{2}^{⊤}(G-\Theta_{0})+\frac{\mu }{2}||H-\Theta_{0}\overset{\sim }{X}|{|}_{F}^{2}+\frac{\mu }{2}||G-\Theta_{0}|{|}_{F}^{2} \end{array}$$

$$\;\;s.t.\;\;{\mathrm{GG}}^{⊤}=I,$$

where the variables $\Lambda_{1}$ and $\Lambda_{2}$ denote the Lagrange multipliers and μ is the regularization parameter.

Under the ADMM optimization framework, the problem (8) can be effectively solved by successively minimizing the object function L for the variables $\Theta_{0},H,G,\Lambda_{1}$, and $\Lambda_{2}$, respectively, when other variables are fixed.

*Optimization with respect to* $\Theta_{0}$*:* The optimization problem of the variable $\Theta_{0}$ can be formulated as follows

(9) $$\underset{\Theta_{0}}{\min}\frac{\beta }{2}(\Theta_{0}\overset{\sim }{X}L{\left(\Theta_{0}\overset{\sim }{X}\right)}^{⊤})+\Lambda_{1}^{⊤}(H-\Theta_{0}\overset{\sim }{X})+\Lambda_{2}^{⊤}(G-\Theta_{0})$$

$$+\frac{\mu }{2}||H-\Theta_{0}\overset{\sim }{X}|{|}_{F}^{2}+\frac{\mu }{2}||G-\Theta_{0}|{|}_{F}^{2},$$

hence its closed-form solution is

(10) $$\Theta_{0}\leftarrow (\mu H{\overset{\sim }{X}}^{⊤}+\Lambda_{1}{\overset{\sim }{X}}^{⊤}+\mu G+\Lambda_{2})\times {\left(\mu \overset{\sim }{X}{\overset{\sim }{X}}^{⊤}+\mu I+\beta \overset{\sim }{X}L{\overset{\sim }{X}}^{⊤}\right)}^{-1}\text{.}$$

*Optimization with respect to* H*:* We estimate the variable H by solving the following optimization problem:

(11) $$\underset{H}{\min}\frac{1}{2}||\overset{\sim }{Y}-P[\Theta_{1},\cdots ,\Theta_{K}]\overset{\sim }{X}-\mathrm{PH}|{|}_{F}^{2}+\Lambda_{1}^{⊤}(H-\Theta_{0}\overset{\sim }{X})+\frac{\mu }{2}||H-\Theta_{0}\overset{\sim }{X}|{|}_{F}^{2}\text{.}$$

For Eq. (11), it is straightforward to derive the analytical solution, i.e.,

(12) $$H\leftarrow {\left(P^{⊤}P+\mu I\right)}^{-1}(P^{⊤}(\overset{\sim }{Y}-P[\Theta_{1},\cdots ,\Theta_{K}]\overset{\sim }{X})+\mu \Theta_{0}\overset{\sim }{X}-\Lambda_{1})\text{.}$$

Algorithm 2 Subprobelm optimization with respect to the variable $\Theta_{0}$

**Table:** 

**Require:**$\overset{\sim }{Y},P,\overset{\sim }{X},L$, and the parameter β, and maxIter.
1. Initialize $\Theta_{0}=0$, the auxiliary variables, e.g., $G=\Lambda_{2}=0,\Lambda_{1}=0$. The regularization parameter is $\mu =10^{-3}$, whose maximal limitation and scaling factor are $\mu_{\max}=10^{6}$ and ρ=1.5, respectively. The iteration starts with t=1 and the tolerate error of the variable is set to $\varepsilon =10^{-6}$.
2. **for** The iteration number t=1 to maxIter**do**
3. Fix other variables and update H using Eq. (12).
4. Fix other variables and update $\Theta_{0}$ using Eq. (10).
5. Fix other variables and update G by the SOC solver, i.e., Eqs. (14), (15).
6. Update Lagrange multipliers $\Lambda_{1}$ and $\Lambda_{2}$ using Eq. (16).
7. Update the regularization parameter using Eq. (17).
8. Check the stopping condition:
9. **if**$||H-\Theta_{0}\overset{\sim }{X}|{|}_{F}<\varepsilon$ and $||G-\Theta_{0}|{|}_{F}<\varepsilon$**then**
10. Stop iteration.
11. **else**
12. t←t+1.
13. **end if**
14. **end for**
**Ensure:** Shared subspace projection $\Theta_{0}$.

*Optimization with respect to* G*:* The optimization problem with the orthogonal constraint for the variable G is

(13) $$\underset{G}{\min}\Lambda_{2}^{⊤}(G-\Theta_{0})+\frac{\mu }{2}||G-\Theta_{0}|{|}_{F}^{2}\;\;s.t.\;\;{\mathrm{GG}}^{⊤}=I,$$

whose solution can be obtained by the means of a well-known solver, i.e., splitting orthogonality constraints (SOC) (Lai and Osher, 2014). The method of SOC solves the orthogonality constrained problem in two steps.

• 1) Singular value decomposition (SVD) is performed on the variable $\Theta_{0}$, i.e.,
  (14) $$\left[U,\Sigma ,V\right]=\mathrm{svd}(\Theta_{0}-\Lambda_{2}/\mu ),$$
  such that $\Theta_{0}-\Lambda_{2}/\mu =U\Sigma V^{⊤}$.
• 2) The orthogonality is satisfied when the variable G is updated by
  (15) $$G\leftarrow {\mathrm{UI}}_{n\times m}V^{⊤}\text{.}$$

*Updating with respect to Lagrange multipliers* $\Lambda_{1}$ *and* $\Lambda_{2}$*:*

(16) $$\Lambda_{1}\leftarrow \Lambda_{1}+\mu (H-\Theta_{0}\overset{\sim }{X}),\;\Lambda_{2}\leftarrow \Lambda_{2}+\mu (G-\Theta_{0})\text{.}$$

*Updating with respect to the regularization parameter* μ*:*

(17) $$\mu \leftarrow \;\min(\rho \mu ,\mu_{\max}),$$

where ρ>1 and $\mu_{\max}$ denote the scaling factor and the maximal value of μ within finite steps, respectively.

The specific optimization procedures for estimating the variable $\Theta_{0}$, i.e., solving the problem (6), are summarized in Algorithm 2.

#### 3.4.3 Learning specific subspace projection matrices — ${\left\{\Theta_{k}\right\}}_{k=1}^{K}$

The solution to the variable $\Theta_{k}$ can be obtained by using the same solver with the problem (6). The only difference lies in that there is no the MA term, i.e., $\frac{\beta }{2}(\Theta_{0}\overset{\sim }{X}L{\left(\Theta_{0}\overset{\sim }{X}\right)}^{⊤})$, in the optimization problem of ${\left\{\Theta_{k}\right\}}_{k=1}^{K}$. As a result, the Algorithm 2 can be directly applied to estimate the variables ${\left\{\Theta_{k}\right\}}_{k=1}^{K}$ as well.

### 3.5 Convergence analysis and computational cost

**Fig. 3:**
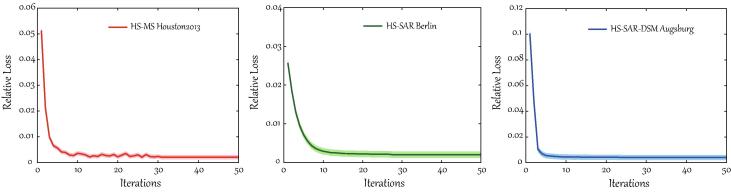
Convergence analysis of the S2FL model on three multimodal RS benchmark datasets: HS-MS *Houston2013*, HS-SAR *Berlin*, and HS-SAR-DSM *Augsburg*.

The global optimization given in Algorithm 1 can be performed by the alternating minimization. The block coordinate descent (BCD) is a commonly-used strategy to solve the problem. The BCD is able to converge well in theory as long as the convexity for each subproblem is met (Bazaraa et al., 2013). The ADMM solver in Algorithm 2 is actually a variant of *inexact* Augmented Lagrange Multiplier (ALM) (Lin et al., 2010), which has been successfully used for solving the multi-block ADMM-based optimization problems (Chen et al., 2016, Deng et al., 2017, Wang et al., 2018). Up to the present, the convergence of the multi-block ADMM in some common cases (e.g., Algorithm 2) has been well studied in various practical cases (Zhou et al., 2016, Xu et al., 2019, Yao et al., 2019), although a *strictly* mathematical proof needs to be further improved and perfected. Moreover, we experimentally record the relative loss of the objective function in each iteration to draw the convergence curves of the proposed S2FL model on three datasets used in the experiment section, as shown in Fig. 3.

Furthermore, it is clear to observe that the overall computational cost of the optimization problem (1) (i.e., our S2FL model) is mainly dominated by classic matrix algebra, such as matrix multiplication and matrix inversion. In detail, the update of the linear regression matrix P exhibits a total complexity with $O(d_{s}^{3}+d_{s}^{2}\mathrm{KN}+d_{s}^{2}C+d_{s}d_{k}K^{2}N+d_{s}\mathrm{CKN})$. For each iteration of Algorithm 2 in solving the subproblem (6), optimizing $\Theta_{0}$ and H generally yields the costs of $O(d_{k}^{3}K^{3}+d_{k}^{2}d_{s}K^{2}+d_{k}^{2}K^{3}N+d_{k}d_{s}K^{2}N+d_{k}K^{3}N^{2})$ and $O(d_{s}^{3}+d_{s}^{2}\mathrm{KN}+d_{s}^{2}C+d_{s}d_{k}\mathrm{KN}+d_{s}d_{k}\mathrm{CK}+d_{k}K^{2}\mathrm{CN}+d_{s}\mathrm{CKN})$, respectively, while updating the variable G requires computing a SVD with the order of cost as $O(\min(d_{k}^{2}d_{s}K^{2},d_{s}^{2}d_{k}K))$. The final update of the specific subspace projection matrices ${\left\{\Theta_{k}\right\}}_{k=1}^{K}$ bears the same complexity as that of the Algorithm 2.

## 4 Experiments

### 4.1 Multimodal benchmark datasets

#### 4.1.1 HS-MS Houston2013 data

The scene consists of HS and MS data, which is a typical homogeneous dataset. The original HS image is available from IEEE GRSS data fusion contest 2013^1^ and has been widely concerned and applied for land cover classification. This image acquires the campus area of the University of Houston, Texas, USA, with 349×1905 pixels and 144 channels covering the spectral range from 0.38μm to 1.05μm. To make full use of high spectral information of the HS image and high spatial information of the MS image, we generate the HS-MS Houston2013 benchmark datasets by degrading the original HS image in spatial and spectral domains. More specifically,

**Spectral degradation:** The low spectral resolution MS image can be obtained by using the spectral response functions (SRFs) of the Sentinel-2 sensor. The resulting MS image is composed of the same size with the original HS image and 8 spectral bands at a ground sampling distance (GSD) of 2.5m.

**Spatial degradation:** The low spatial resolution HS image with a 10m GSD is generated by the means of the bilinear interpolation. To meet the pixel-to-pixel correspondences between HS and MS images, the degraded HS image is re-upsampled to the size of the MS image, i.e., 349×1905, by using the nearest neighbor interpolation.

**Fig. 4:**
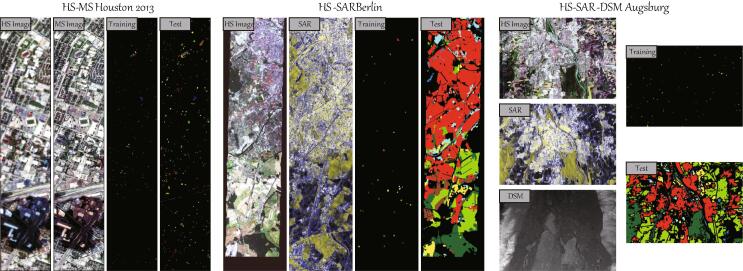
False-color visualization as well as the spatial distribution of training and test samples of three multimodal RS benchmark datasets: HS-MS *Houston2013*, HS-SAR *Berlin*, and HS-SAR-DSM *Augsburg*.

Table 2 lists the types of ground objects and the number of training and test samples used for the classification task in the HS-MS Houston2013 datasets, and correspondingly Fig. 4 visualizes the false-color images and the distribute of training and test sets.

**Table 2:** Description of the investigated HS-MS Houston2013 datasets, including the types of ground objects and the corresponding number of training and test samples.

No.	Ground Object Name	Training Set	Test Set
1	Healthy Grass	198	1053
2	Stressed Grass	190	1064
3	Synthetic Grass	192	505
4	Tree	188	1056
5	Soil	186	1056
6	Water	182	143
7	Residential	196	1072
8	Commercial	191	1053
9	Road	193	1059
10	Highway	191	1036
11	Railway	181	1054
12	Parking Lot1	192	1041
13	Parking Lot2	184	285
14	Tennis Court	181	247
15	Running Track	187	473

	Total	2832	12197

#### 4.1.2 HS-SAR Berlin data

The simulated EnMAP data synthesized based on the HyMap HS data graphically describes the Berlin urban and its rural neighboring area at 30m GSD, which can be freely downloaded from the website^2^. In detail, there are 797×220 pixels in this scene, where 244 spectral bands are given in the wavelength range of 0.4μm to 2.5μm. More details can be found in (Okujeni et al., 2016). To get a corresponding SAR data of the same region, we download a Sentinel-1 dual-Pol (VV-VH) single look complex (SLC) product from ESA. The product is collected under the Interferometric Wide (IW) swath mode. With the help of ESA toolbox SNAP^3^, we build up a pre-processing workflow to prepare the SLC product as an analysis-ready SAR image. The workflow includes apply orbit profile, radiometric calibration, deburst, speckle reduction, terrain correction, and region-of-interest extraction. The analysis-ready SAR image used in this paper is geocoded in UTM/WGS84 coordinate system, and it is spatially averaged while applying speckle reduction and saved in 2×2 PolSAR covariance matrix. Since the azimuth resolution of the Sentinel-1 data is approximately 13m, the processed SAR image has a 13.89m GSD and consists of 1723×476 pixels. Similarly to the first datasets, the nearest neighbor interpolation is performed on the HS image, enabling the same image size with the SAR data.

The ground reference data for land cover classification is generated by using the OpenStreetMap data (Haklay and Weber, 2008), where the information regarding the training and test sets is shown in Fig. 4 and Table 3.

**Table 3:** Description of the investigated HS-SAR Berlin datasets, including the types of ground objects and the corresponding number of training and test samples.

No.	Ground Object Name	Training Set	Test Set
1	Forest	443	54511
2	Residential Area	423	268219
3	Industrial Area	499	19067
4	Low Plants	376	58906
5	Soil	331	17095
6	Allotment	280	13025
7	Commercial Area	298	24526
8	Water	170	6502

	Total	2820	461851

#### 4.1.3 HS-SAR-DSM augsburg data

This dataset consists of three different data sources, including a spaceborne HS image, a dual-Pol PolSAR image, and a DSM image, where HS and DSM data are acquired by DAS-EOC, DLR, and the PolSAR data are collected from the Sentinel-1 platform, over the city of Augsburg, Germany. They are collected by the HySpex sensor (Baumgartner et al., 2012), the Sentinel-1 sensor, and the DLR-3 K system (Kurz et al., 2011), respectively. To evaluate the performance of multimodal fusion classification effectively, we downsample the spatial resolution of all images to a unified 30m GSD. The scene comprises of 332×485 pixels and 180 spectral bands ranging from 0.4μm to 2.5μm for the HS image, 1 band for the DSM image, and 4 features from the dual-Pol (VV-VH) SAR image. Note that the SAR data is preprocessed in the same way as the SAR component in HS-SAR Berlin Data. The 4 features are VV intensity, VH intensity, the real part and the imaginary part of the off-diagonal element of the PolSAR covariance matrix. The ground reference data is generated from the OpenStreetMap data. Detailed information regarding the training and test sets is shown in Fig. 4 and Table 4.

**Table 4:** Description of the investigated HS-SAR Augsburg datasets, including the types of ground objects and the corresponding number of training and test samples.

No.	Ground Object Name	Training Set	Test Set
1	Forest	146	13361
2	Residential Area	264	30065
3	Industrial Area	21	3830
4	Low Plants	248	26609
5	Allotment	52	523
6	Commercial Area	7	1638
7	Water	23	1507

	Total	761	77533

### 4.2 Experimental setup and preparation

#### 4.2.1 Evaluation metrics

In the experiments, land cover classification is regarded as a potential application to evaluate the quality of the multimodal feature representations learned by the proposed S2FL model. More specifically, we compute three widely-used criteria, i.e., *Overall Accuracy (OA)*, *Average Accuracy (AA)*, and *Kappa Coefficient* (κ) to make a quantitative performance comparison using a nearest neighbor (NN) classifier. More specifically, we first apply the proposed S2FL model to extract or learn the multimodal feature representations and then feed them into a classifier (NN in our case). The main reason to select the NN classifier can be clarified by the fact that we expect to see the performance gain owing to the learned features obtained by our proposed method rather than those advanced classifiers, e.g., support vector machine (SVM), random forest (RF), deep learning-based classifiers, etc.

#### 4.2.2 Comparison with state-of-the-art MFL models

Several state-of-the-art MFL algorithms related to land cover classification of multimodal RS images are selected as competitors, compared to the proposed S2FL model. They are joint dimensionality reduction based on principal components analysis (JDR-PCA)(Martínez and Kak, 2001), supervised manifold alignment (SMA) (Wang and Mahadevan, 2011), unsupervised manifold alignment (USMA) (Liao et al., 2016), common subspace learning ($\ell_{2}$-CoSpace) (Hong et al., 2019a), $\ell_{1}$-CoSpace (Hong et al., 2020a), as well as single modalities, e.g., HS, MS, SAR, DSM, and their simple concatenation, e.g., HS + MS, HS + SAR, HS + SAR + DSM.

#### 4.2.3 Implementation details

The algorithm performance, to a great extent, depends on the parameter tuning, e.g., regularization parameters in CoSpace and our S2FL. As a result, selecting a proper range for these parameters is of great significance in practical applications. For this reason, a 10-fold cross validation is conducted on the training set to determine the parameter combination of different methods. These parameters are tuned in a given range to maximize the classification accuracy. For example, the number of nearest neighbors (*q*) and the parameter σ for computing the adjacency matrix W in USMA and S2FL can be selected from {5,10,…,50} and $\left\{10^{-2},10^{-1},10^{0},10^{1},10^{2}\right\}$, respectively. The regularization parameters, e.g., α and β in CoSpace and S2FL, can be determined in the range of $\left\{10^{-3},10^{-2},10^{-1},10^{0},10^{1},10^{2}\right\}$. Moreover, the feature dimension is also a key parameter, which has great effects on the quality of final learned feature representations. For that, the optimal feature dimension (*d*) can be found out from 5 to 50 at a 5 interval, according to the best classification performance on the training set.

It should be noted, however, that the DSM image only holds one feature band in the *Augsburg* datasets, hence attribute profiles are first extracted to fully utilize the spatial information of the DSM image, extending the feature bands to 3, before performing feature learning and classification.

### 4.3 Results and analysis on Houston2013 datasets

Table 5 lists the quantitative classification results of compared feature learning methods on HS-MS *Houston2013* datasets, including *OA*, *AA*, κ, and the accuracy for each class. Overall, the joint use of multiple modalities (e.g., HS + MS) obviously performs better than single modalities in three main indices, i.e., *OA*, *AA*, and κ. The classification accuracies obtained by JDR-PCA are basically same to those jointly using HS and MS data. For MA-based approaches (e.g., USMA, SMA), they fail to classify the materials well, due to their sensitivity to various complex noises. This indirectly leads to relatively poor performance, compared to PCA and HS + MS. Owing to the use of label (supervised) information, the classification accuracy of SMA is higher than that of USMA, bringing an increase of about 2% points OA. Moreover, the joint learning-based group, e.g., $\ell_{2}$-CoSpace, $\ell_{1}$-CoSpace, and S2FL, dramatically outperforms other competitors, either in main indices (*OA*, *AA*, and κ) or in the accuracy for the majority of categories. The performance of $\ell_{1}$-CoSpace is superior to that of $\ell_{2}$-CoSpace (approximately 2% points improvement), since the feature selection strategy is performed in $\ell_{1}$-CoSpace by the means of sparsity-promoting $\ell_{1}$-norm term. More remarkably, our proposed S2FL model can obtain a higher classification result with around 5% points gain in *OA* on the basis of $\ell_{1}$-CoSpace that ranks the second place. In addition, S2FL also plays a dominated role in the classification accuracy of each class. That is, S2FL is capable of achieving the best classification performance in many categories (e.g., *Soil*, *Commercial*, *Road*, *Highway*, *Railway*, *Tennis Court*, and *Running Track*), which demonstrates its effectiveness and superiority in the land cover classification task.

**Table 5:** Quantitative results of different compared approaches in terms of OA, AA, and κ as well as the accuracy for each class on HS-MS *Houston2013* datasets using NN classifier, where the parameters are determined by cross-validation on the training sets. The best one is shown in bold.

Method	MS	HS	HS + MS	JDR-PCA	USMA	SMA	$\ell_{2}$-CoSpace	$\ell_{1}$-CoSpace	S2FL
Parameters	–	–	–	*d*	q,σ,d	*d*	α,β,d	α,β,d	α,β,d
				20	10,1,30	30	1,0.01,30	0.1,0.1,30	0.01,0.1,30
Healthy Grass	**82.53**	76.64	79.39	79.39	82.05	81.01	81.10	80.15	80.06
Stressed Grass	81.95	74.25	76.60	76.60	80.83	**84.68**	83.46	83.36	84.49
Synthetic Grass	98.81	91.09	93.47	93.07	99.01	97.82	97.03	**99.41**	98.02
Trees	**91.29**	70.17	81.72	81.72	90.81	88.45	88.54	89.77	87.31
Soil	96.31	98.48	98.48	98.48	94.89	97.25	98.39	99.72	**100.00**
Water	**98.60**	77.62	81.12	81.12	95.10	83.22	95.10	81.82	83.22
Residential	72.01	67.07	73.51	73.41	70.34	74.72	**77.99**	71.08	73.32
Commercial	34.00	41.22	41.31	41.31	34.28	43.11	44.35	64.10	**74.84**
Road	63.74	60.15	63.36	63.36	67.23	62.13	66.19	65.91	**78.38**
Highway	43.44	44.79	46.14	46.14	41.80	40.64	58.01	60.81	**83.30**
Railway	64.52	57.12	57.50	57.50	57.21	51.71	70.59	79.70	**81.69**
Parking Lot 1	46.88	79.54	75.50	75.41	45.24	62.73	87.80	83.57	**95.10**
Parking Lot 2	52.28	70.53	**73.33**	**73.33**	38.25	60.00	67.02	70.18	72.63
Tennis Court	96.36	88.26	97.57	97.57	95.55	98.79	98.79	**100.00**	**100.00**
Running Track	98.52	82.24	90.49	90.49	97.89	98.31	**99.37**	98.94	**99.37**

OA (%)	70.82	69.21	72.02	71.98	69.39	71.66	77.97	79.86	**85.07**
AA (%)	74.75	71.94	75.30	75.26	72.70	74.97	80.91	81.90	**86.11**
κ	0.6866	0.6684	0.6984	0.6980	0.6705	0.6929	0.7629	0.7814	**0.8378**

**Table 6:** Quantitative results of different compared approaches in terms of OA, AA, and κ as well as the accuracy for each class on HS-SAR *Berlin* datasets using NN classifier, where the parameters are determined by cross-validation on the training sets. The best one is shown in bold.

Method	SAR	HS	HS + SAR	JDR-PCA	USMA	SMA	$\ell_{2}$-CoSpace	$\ell_{1}$-CoSpace	S2FL
Parameters	–	–	–	*d*	q,σ,d	*d*	α,β,d	α,β,d	α,β,d
				10	10,1,10	10	1,0.01,10	0.1,1,10	0.01,1,10
Forest	24.47	71.45	73.57	73.56	62.19	57.34	79.70	82.94	**83.30**
Residential Area	19.86	41.19	42.93	42.81	43.29	50.53	50.51	52.75	**57.39**
Industrial Area	25.53	40.37	38.72	38.66	27.97	43.46	**49.63**	44.71	48.53
Low Plants	31.79	68.07	69.23	68.03	63.46	50.03	69.27	76.54	**77.16**
Soil	31.61	81.05	81.01	81.01	79.02	73.60	70.30	81.92	**83.84**
Allotment	13.32	63.89	62.89	61.90	48.33	52.69	**66.23**	55.36	57.05
Commercial Area	14.96	35.31	36.83	**36.94**	23.51	26.59	32.02	29.64	31.02
Water	7.91	65.76	67.13	**67.21**	47.34	65.20	61.80	45.36	61.57

OA (%)	21.98	50.30	51.71	51.47	47.93	50.83	56.67	58.84	**62.23**
AA (%)	21.18	58.39	59.04	58.77	49.39	52.43	59.93	58.65	**62.48**
κ	0.0759	0.3709	0.3838	0.3809	0.3243	0.3528	0.4306	0.4571	**0.4877**

**Fig. 5:**
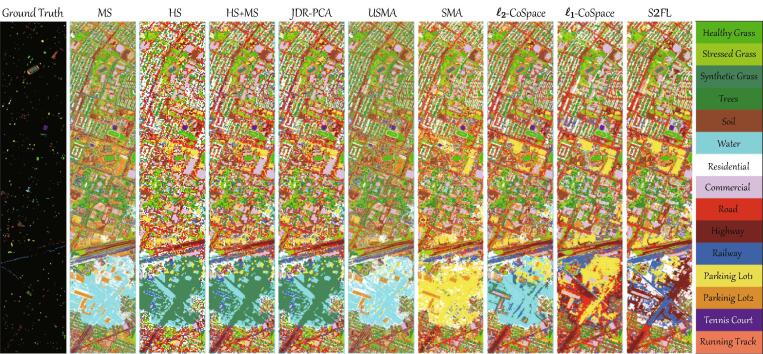
Classification maps obtained by different MFL algorithms on the *Houston2013* datasets.

Visually, Fig. 5 shows the integral classification maps of considered compared algorithms in the given scene. There is a basically identical trend in both quantitative and qualitative comparisons (between Fig. 5 and Table 5). It is worth noting that the classification map obtained by the proposed S2FL model has not only more structurally geometric information for those man-made materials, e.g., *Residential*, *Commercial*, *Parking Lot*, etc., but also more detailed textural information, e.g., for the ground objects of *Grass* and *Tree*.

#### 4.3.1 Parameter analysis of S2FL model

**Fig. 6:**
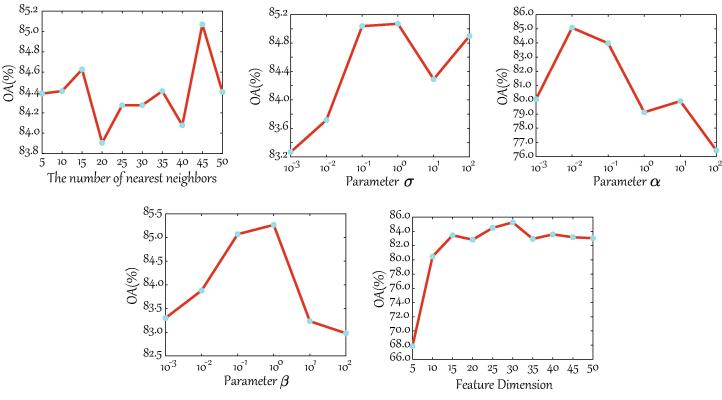
Parameter sensitivity analysis of S2FL model conducted on the HS-MS *Houston2013* datasets, e.g., the number of nearest neighbors and σ in Eq. (2), the regularization parameters α and β in Eq. (1), and the feature dimension.

Parameter (or model) selection is the key factor to wield significant influence over the performance gain of the feature learning method. It is necessary, therefore, to discuss and analyze the parameter sensitivity. There are five main parameters, i.e., the number of nearest neighbors (*q*) and the parameter σ in Eq. (2), the regularization parameters (α and β) in Eq. (1), and the feature dimension (*d*), in the S2FL model. As shown in Fig. 6, the regularization parameter α and the feature dimension *d* yield a higher change in the term of *OA* with different input values, compared to other three parameters. Moreover, varying the number of nearest neighbors *q* is relatively insensitive to the classification accuracy, while the parameters σ and β can moderately control the change of *OA*. From the overall perspectives, the *OA* value remains stable as long as these parameters are selected in a proper range. The optimal parameter combination is $\left(q,\sigma ,\alpha ,\beta ,d)=(45,10^{0},10^{-2},10^{0},30\right)$, which is basically identical to that obtained by cross-validation on the training set. This, to some extent, demonstrates the acceptable practicability of the proposed S2FL model in the real application.

### 4.4 Ablation analysis of the proposed S2FL model

To verify the effectiveness of the proposed idea (i.e., shared and specific feature disentangling) in the S2FL model, we investigate the performance gain with the use of different components, i.e., only using modality-shared features (obtained via $\Theta_{0}$), only using modality-specific features (obtained via $\Theta_{k}$). The quantitative results in terms of *OA*, *AA*, and κ indices on the *Houston2013* datasets are reported in Table 7. More specifically, the S2FL’s results only using one modality (e.g., HS, MS)^4^ are reported, which is better than those without feature learning (the first two rows in Table 7). We also consider the case of only using shared features or only using specific features in our S2FL model for land cover classification. As can be seen from Table 7, the performance of S2FL by considering multimodal input is superior to that of only using single modalities, while the modality-specific information is more important than the modality-shared information (*OA*: 83.11 vs 78.77) in the classification task. Moreover, we found that the performance happens a dramatic degradation without the orthogonal constraint in the S2FL model. Not unexpectedly, our S2FL with a joint combination of shared and specific features achieves the best results.

**Table 7:** Ablation analysis of the proposed S2FL model on the Houston2013 datasets.

Model	HS	MS	Orthogonality	Shared	Specific	OA (%)	AA (%)	κ
–						69.21	71.94	0.6684
–						70.82	74.75	0.6866
S2FL						76.50	78.80	0.7456
S2FL						72.74	76.38	0.7070
S2FL						78.77	81.59	0.7697
S2FL						83.11	84.52	0.8166
S2FL						66.11	69.82	0.6321
S2FL						**85.07**	**86.01**	**0.8378**

### 4.5 Cross-modality experiments: A special case of multi-modality

Take the bi-modality as an example, cross-modality learning (CML) for simplicity refers to that training a model using two modalities and one modality is absent in the testing phase, or *vice versa* (only one modality is available for training and bi-modality for testing) (Ngiam et al., 2011). Such a CML problem that exists widely in various RS tasks is more applicable to real-world cases. n recent years, there have been some works proposed to investigate the CML issue and applied for land cover classification. More details regarding the CML’s setting can refer to (Hong et al., 2019a, Hong et al., 2019c). Table 8 lists the quantitative comparison between the single modalities and the CML’s cases using our S2FL. There is a basically consistent trend in both HS and MS data. That is, the classification accuracies of directly using the original spectral features (either HS or MS) are lower than those using learned features (e.g., via S2FL). In addition, the features learned by the S2FL-CML setting can better classify the land cover materials compared to those directly learning features from the single modalities (e.g., S2FL-HS or S2FL-MS), showing the effectiveness of the proposed S2FL model for both cases of multi-modality learning and the CML.

**Table 8:** Quantitative comparison using the S2FL model under the cross-modality setting. “Only HS (MS)” means directly using original HS (MS) data as the features, “S2FL-HS (MS)” means the single modality feature learning (e.g., HS or MS), and “S2FL-CML-HS (MS)” means the CML setting, i.e., training on HS-MS data and only HS (MS) data are available in the testing phase.

Method	Only HS	S2FL-HS	S2FL-CML-HS	Only MS	S2FL-MS	S2FL-CML-MS
OA (%)	69.21	76.50	80.86	70.01	72.74	77.01
AA (%)	71.94	78.80	82.21	80.46	76.38	80.46
κ	0.6684	0.7456	0.7922	75.09	0.7070	0.7509

### 4.6 Results and analysis on Berlin datasets

Unlike the homogeneous HS-MS data, the heterogeneity between HS and SAR data remains challenging in the feature learning and fusion. Therefore, the quantitative comparison conducted on the *Berlin* datasets (see Table 6) becomes significantly meaningful for the performance assessment of MFL models. As can be seen in Table 6, the heterogeneous datasets are challenging and difficult to perform the land cover classification, yielding a sharp decrease in classification performance compared to results on the *Houston2013* datasets. Nevertheless, the whole trend between different algorithms is similar. The classification accuracy using the concatenation of HS and SAR data is still higher than that only using single modalities. PCA-based joint feature learning obtains a basically same result with HS + SAR’s. Similarly, USMA and SMA fail to align the heterogeneous data well. The reasons are twofold: the sensitivity to the noises and only considering the shared component representations across modalities. The CoSpace family, i.e., $\ell_{2}$-CoSpace and $\ell_{1}$-CoSpace, is robust to the noise effects by learning the latent subspace to bride the multimodal data and label information, bringing increments of 5.84% and 8.01% points *OA*, respectively, on the basis of SMA. Not unexpectedly, our S2FL method achieves the best performance with further improvement of 3.39%, 3.83%, and 3.06% points with respect to *OA*, *AA*, and κ over $\ell_{1}$-CoSpace. Additionally, the S2FL model can also obtain desirable classification accuracy for each class in comparison with other approaches.

**Fig. 7:**
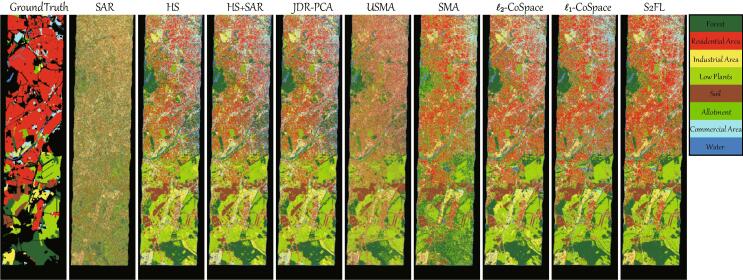
Classification maps obtained by different MFL algorithms on the *Berlin* datasets.

Fig. 7 shares a similar visual comparison with quantitative results. Only using SAR data yields a poor classification map with extensive noisy points. Notably, our method is capable of fully blending the HS and SAR information by the means of the interpretable shared and specific feature learning mechanism, thereby reducing the noisy pixels and generating more smooth classification maps. Particularly for those ground objects that hold rich texture information, e.g., *Forest*, *Low Plants*, the S2FL model tends to capture their subtle differences against noises by the means of specific information of each modality. Furthermore, the learned common components can depict the structural information, thereby further identifying the materials of *Residential* and *Commercial* effectively.

### 4.7 Results and analysis on Augsburg datasets

We further investigate the generalization ability and effectiveness of the proposed S2FL model in the case of three-modality data, i.e., HS, SAR, and DSM. Table 9 details the classification results of different compared algorithms. Generally, there is an obvious improvement (around 4%) in *OA* when jointly using three modalities, compared to that using HS, SAR, and DSM independently. When the number of considered modalities increases to 3, the performance of those MA-based models dramatically declines, especially USMA that is a lack of label guidance. It is worth noted, however, that the CoSpace-based approaches modeled by either $\ell_{2}$-norm or $\ell_{1}$-norm, can be effectively extended to the case of three modalities, yielding significant improvement in classification accuracies. The feature selection works well in handling the issue of multiple heterogeneous data. That is, the classification result of the $\ell_{1}$-CoSpace is higher than that of $\ell_{2}$-CoSpace, which increases by 5.32% points *OA*. As excepted, the S2FL performs better than $\ell_{1}$-CoSpace with an increase of approximately 2 percentage points *OA*. More importantly, the *AA* value obtained by the S2FL is far higher than other competitors, where almost of each class can achieve the highest classification results. This, to a great extent, shows the superiority of our proposed shared and specific learning strategy (i.e., S2FL model).

**Table 9:** Quantitative results of different compared approaches in terms of OA, AA, and κ as well as the accuracy for each class on HS-SAR-DSM *Augsburg* datasets using NN classifier, where the parameters are determined by cross-validation on the training sets. The best one is shown in bold.

Method	SAR	DSM	HS	HS + SAR + DSM	JDR-PCA	USMA	SMA	$\ell_{2}$-CoSpace	$\ell_{1}$-CoSpace	S2FL
Parameters	–	–	–	–	*d*	q,σ,d	*d*	α,β,d	α,β,d	α,β,d
					20	10,1,20	20	1,0.1,20	0.1,0.1,20	0.1,0.01,20
Forest	44.40	53.18	81.75	88.39	88.32	65.53	81.94	82.04	87.79	**88.80**
Residential Area	70.10	66.70	77.01	82.91	82.93	79.14	73.43	83.52	**88.32**	86.36
Industrial Area	13.79	11.83	26.84	22.38	22.27	21.85	24.18	34.99	32.64	**38.90**
Low Plants	61.55	55.06	67.67	69.24	69.06	56.36	81.12	76.67	88.44	**90.53**
Allotment	9.18	25.24	46.65	55.45	55.07	44.17	22.75	52.77	45.89	**68.64**
Commercial Area	8.30	3.48	12.21	11.29	11.29	5.62	8.79	**14.22**	12.09	8.97
Water	6.97	5.84	43.20	47.38	47.38	17.98	32.85	**48.71**	17.65	47.45

OA (%)	57.01	54.87	69.91	73.78	73.71	63.17	72.61	76.17	81.49	**83.36**
AA (%)	30.61	31.62	50.76	53.86	53.76	41.52	46.44	56.13	53.56	**61.38**
κ	0.3974	0.3571	0.5742	0.6252	0.6241	0.4776	0.6046	0.6643	0.7327	**0.7626**

**Fig. 8:**
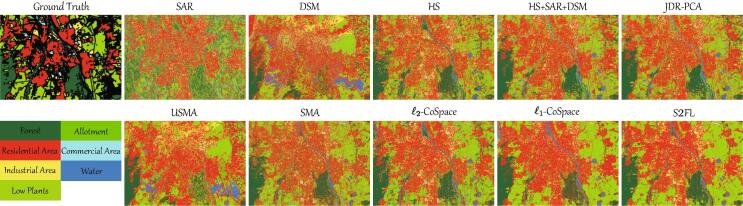
Classification maps obtained by different MFL algorithms on the *Augsburg* datasets.

Furthermore, the classification maps shown in Fig. 8 also give a strong support to the aforementioned conclusion. The S2FL by the means of multiple modality data can obtain more realistic material identification in land cover mapping. In particular, the materials, e.g., *Forest*, *Residential Area*, *Low Plants*, are classified in more smooth fashion, showing semantically meaningful structure.

#### 4.7.1 Ablation study on the use of multiple modalities

To further verify the effectiveness and superiority of the proposed S2FL model in multimodal RS data feature learning and fusion, we will investigate the performance gain by using different combinations of multiple modalities. More specifically, three high-performance MFL algorithms, i.e., $\ell_{2}$-CoSpace, $\ell_{1}$-CoSpace, S2FL, are used for quantitative comparison on the HS-SAR-DSM *Augsburg* datasets, as detailed in Table 10. On the whole, there are several important and intuitive conclusions, which can be summarized as follows:

• The joint exploitation of multiple modalities can break the performance bottleneck in land cover classification. For example, the HS + SAR + DSM can usually obtain better classification results than the only use of two modalities.
• Characterized by rich spectral information, the HS image tends to identify the materials at a more accurate level compared to SAR and DSM.
• The HS + SAR results obtained by $\ell_{2}$-CoSpace are even slightly better than those of HS + SAR + DSM. This indicates that the $\ell_{2}$-CoSpace method fails to better fuse the multimodal information to some extent when the number of modalities increases.
• Feature selection guided by sparsity-promoting $\ell_{1}$-norm is an effective strategy for MFL. The resulting $\ell_{1}$-CoSpace observably outperforms $\ell_{2}$-CoSpace in different modality combinations.
• By decoupling the multimodal data into shared and specific components, S2FL is capable of better learning feature representations of multimodal data (*cf.* $\ell_{2}$-CoSpace and $\ell_{1}$-CoSpace), further yielding higher classification performance in either two modalities or three modalities.

**Table 10:** Ablation study for different modality combinations by using three high-performance MFL algorithms (i.e., $\ell_{2}$-CoSpace, $\ell_{1}$-CoSpace, S2FL). The best one is shown in bold.

Modality Combination	Methods	OA (%)	AA (%)	κ
SAR + DSM	$\ell_{2}$-CoSpace	68.14	39.29	0.5490
	$\ell_{1}$-CoSpace	69.32	39.50	0.5649
	S2FL	**69.49**	**40.15**	**0.5674**
HS + DSM	$\ell_{2}$-CoSpace	70.79	53.41	0.5887
	$\ell_{1}$-CoSpace	**81.17**	52.75	0.7268
	S2FL	81.11	**59.09**	**0.7313**
HS + SAR	$\ell_{2}$-CoSpace	76.82	54.55	0.6722
	$\ell_{1}$-CoSpace	80.96	51.26	0.7243
	S2FL	**82.34**	**59.92**	**0.7493**
HS + SAR + DSM	$\ell_{2}$-CoSpace	76.17	56.13	0.6643
	$\ell_{1}$-CoSpace	81.49	53.56	0.7327
	S2FL	**83.36**	**61.38**	**0.7626**

It is worth noting that currently-developed DL-based approaches have shown great potential in the fusion and representation learning of multimodal RS data, yet these methods inevitably suffer from various possible performance degradation. However, facing these problems, the proposed S2FL model could, to a great extent, offer capabilities that DL methods do not provide in the aspects of robustness, interpretability, and sensitivity to the training set size.

## 5 Conclusion

Land cover classification has long been considered as a main research topic in the RS and geoscience community. As a crucial step, feature extraction has been paid much attention by researchers. However, the feature representation ability extracted from only single RS data resources remains limited. Fortunately, the rapid development of RS imaging techniques makes multimodal RS data available on a large scale. To speed up the development of multimodal RS data processing and analysis, we in this paper aim at opening three multimodal RS benchmark datasets, they are homogeneous HS-MS *Houston2013*, heterogeneous HS-SAR *Berlin*, and three-modality HS-SAR-DSM *Augsburg* datasets. Further, we also propose a novel MFL model, called S2FL, yielding more discriminative and compact feature blending by learning shared-modality and specific-modality representations. By comparing with previously-proposed advanced MFL methods, the S2FL model obtains the best classification performance on the three datasets, which is obviously superior to other competitors. We will open the three potential benchmark datasets and the MFL toolbox including newly-proposed S2FL model, contributing to the RS and information fusion community. In future work, we would like to further extend these datasets to a larger scale and also develop the corresponding feature learning models, e.g., based on more powerful deep learning techniques by embedding more interpretable knowledge or priors to guide the network optimization in the multimodal feature learning task.

## Declaration of Competing Interest

The authors declare that they have no known competing financial interests or personal relationships that could have appeared to influence the work reported in this paper.

## Footnotes

1 http://www.grss-ieee.org/community/technical-committees/data-fusion/2013-ieee-grss-data-fusion-contest/.

2 http://doi.org/10.5880/enmap.2016.002.

3 https://step.esa.int/main/toolboxes/snap/.

4 In this case, our S2FL is reduced to the single modality feature learning model, i.e., only one $\Theta_{k}$ for either HS or MS.
